# Fabrication of Superhydrophobic Gully-Structured Surfaces by Femtosecond Laser and Imprinting for High-Efficiency Self-Cleaning Rain Collection

**DOI:** 10.1021/acs.langmuir.1c03488

**Published:** 2022-02-16

**Authors:** Gan Yuan, Yu Liu, Fei Xie, Chunlei Guo, Chi-Vinh Ngo, Wei Li

**Affiliations:** † GPL Photonics Lab, State Key Laboratory of Applied Optics, Changchun Institute of Optics, Fine Mechanics and Physics, Chinese Academy of Sciences, 130033 Changchun, China; ‡ University of Chinese Academy of Sciences, 100049 Beijing, China; § The Institute of Optics, University of Rochester, Rochester, New York 14627, United States

## Abstract

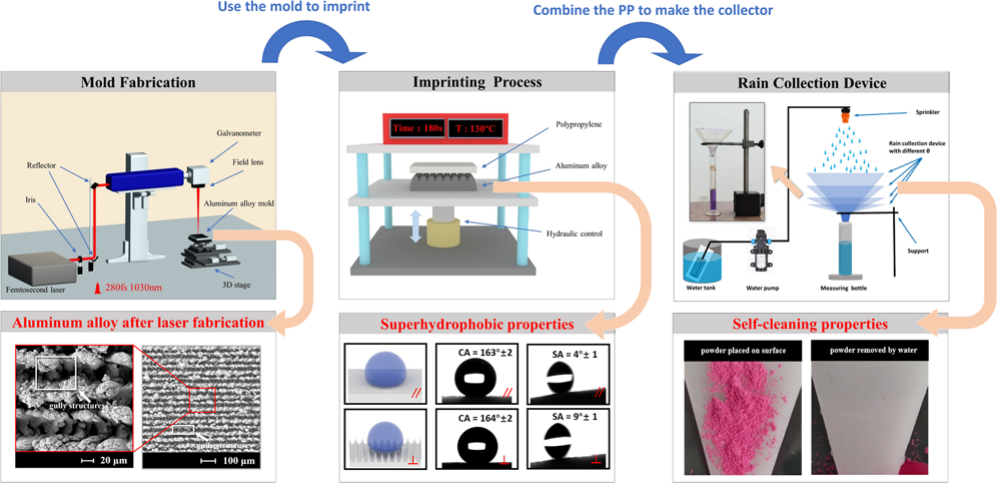

Freshwater is considered an essential need for humanity. Moreover, it is important to collect and make full use of rainwater. This work utilizes a femtosecond laser to fabricate micro-nanostructures on aluminum alloy substrates as molds. Then, the structures are imprinted on cheap and wildly used polypropylene (PP) materials. The just-imprinted PP surfaces with instinctive surface energy and replicated micro-nanostructures have an excellent superhydrophobic property with contact angles greater than 160° and anisotropic sliding angles smaller than 5° in parallel directions and smaller than 10° in the vertical directions. A small-scale rain collection device formed by a combination of the superhydrophobic PP surfaces is used to investigate the effects of the rain collection efficiency and total surface area relating to manufacturing cost. The rain collection device formed by the imprinted PP surfaces has high rain collection efficiency in terms of the volume of the collected water per square centimeter. For the light rain, the rain collection efficiency can reach an approximated maximum of 90%, more than 100% efficiency improvement of the device formed by flat PP surfaces in some cases. Therefore, the rain collection device is helpful in collecting water from rains in arid areas.

## 1 Introduction

Water is one of the Earth’s most abundant resources, 97% of it is saline that covers three-quarters of the Earth’s surface, and only 3% is freshwater, which is essential for daily human life.^1^ While expecting 2.5% is blocked in the polar ice caps, glaciers, and atmosphere, only 0.5% of water can be used for humans in the form of river water and groundwater.^2^ Moreover, about 30% of the world’s population lacks access to clean water sources for fundamental sanitation needs. As a result, more than half of the global population lives in absolute water scarcity or severe water stress regions.^1^

Rain as part of freshwater can be collected for thousands of years. However, the rain collection process still suffers from several problems like attachment of water to the rain collector surface, resulting in a decline in the rain collection efficiency.^3^ Typical ways to collect rain use a roof coated with lead paint or fittings, which causes potential pollution by toxic chemicals.^3,4^ More recently, femtosecond (fs) laser fabrication has been used as a new manufacturing method for developing functional surfaces in water collection applications.^5^ Compared to conventional machining processes, femtosecond laser fabrication is more precise processing for multifunctional surfaces, including micro-nanoscale structures without mechanical stress, and multiple materials such as semiconductors, metal, plastic, glass, ceramics, and graphene.^6−22^ At the same time, femtosecond laser fabrication is also uncomplicated processing and is available for industry.^5^ Moreover, the femtosecond laser is used for fabricating mircro-nanoscale structures with the desired photonic, thermal, and superhydrophilic or superhydrophobic properties.^23−29^

The superhydrophobic surface with different patterns is typically used for water collection or fog collection.^29−31^ However, there is no research on making a three-dimensional (3D) trapezoid device with superhydrophobic surfaces for rain collections, especially for light rains. In addition, based on the rain data of different relative drought areas like China Guizhou, the Middle East, southern Britain, Ethiopia, and India, simulated rains are made to test the rain collection efficiency.^32−36^ Furthermore, exceptionally for the light rain and the beginning of the rain, the water is attached to the flat surface, and only little water can be collected in water tanks because most of the water stays on the surface and then evaporates. However, if the surface has superhydrophobic properties, the rain easily falls from the surfaces to water tanks, improving the collection efficiency. Moreover, the superhydrophobic surface also has a self-cleaning property. It means that the surfaces of the rain collection device can keep clean, which is very useful for rain collection. This work can also combine with night-time harvesting and radiative cooling in the future.^37,38^

In this work, we employed a femtosecond laser ablation and molding process to produce a rain collection device. After the laser ablation process, the micro-nanostructures on the molds can be replicated on cheap, stable, and common polypropylene (PP) polymers to produce superhydrophobic surfaces, followed by a combination of these surfaces to make a small device for highly efficient rain collection. For example, in the case of 1.6 mm rain, the rain collection efficiency can reach about 90% for the device with θ = 30°. Moreover, the rain collection efficiency of the imprinted surfaces is increased more than one time compared with the flat PP surfaces at the rain of 1.6 mm and θ = 30°. Finally, different setups of the rain collection device are produced by changing the slopes of the device’s sidewalls to investigate the rain collection efficiencies and the total surface areas related to manufacturing cost for proposing an optimal device.

## 2 Experimental Methods

### 2.1 Sample Preparation

The samples in this work were the substrates of 50 × 50 mm^2^ size and 5 mm thickness made of the 6061 aluminum alloys (Shenzhen Zhibao Metal Products Co., Ltd. China). The PP (Dongguan Kai’an Plastic Material Co., Ltd. China) plates were cut into 10 × 10 mm^2^ with 1 mm thickness and different trapezoid sheets of 2 mm thickness, as shown in Table 1. A saturated solution of stearic acid (Aladdin) in ethanol (Beijing Chemical Works) is used for the demolding process.

**Table 1:**
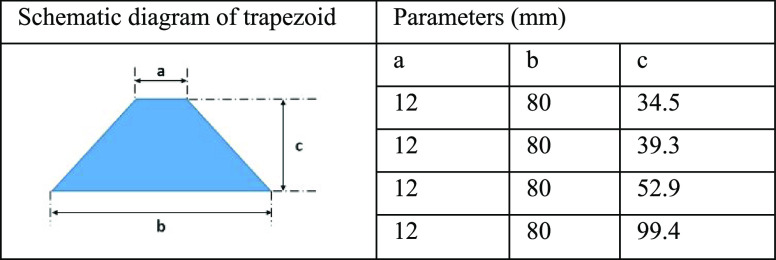
Different Sizes of Trapezoid PP

### 2.2 Surface Characterization

A scanning electron microscope (SEM) (ZEISS, Auriga-45-06) is used to characterize the aluminum alloy molds ablated by the fs laser and the imprinted PP surfaces. The surface morphologies are analyzed by a laser confocal microscope (Keyence, VK-X1000). The contact angles and sliding angles are measured by a contact angle meter (POWEREACH, JC2000D3) to characterize the wettability of the samples.

### 2.3 FS Laser Setup and Fabrication

**Figure 1:**
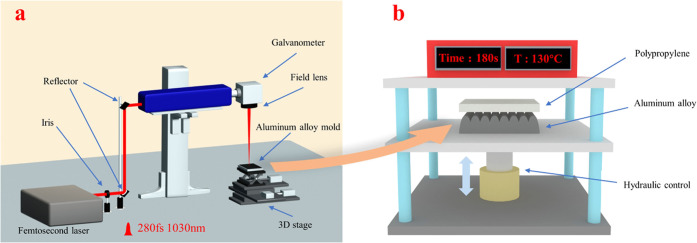
(a) Laser (1030 nm fs) setup to fabricate the aluminum alloy mold with micro-nanostructures. (b) Imprinting setup to replicate the structures from the aluminum alloy to the polypropylene.

The fs laser power of 5 W and the frequency of 100 kHz are used to make micro-nanostructures on the aluminum alloy surfaces. A YAG fs laser (PHAROS-Light Conversion) with a 280 fs 1030 nm laser beam is used to produce structures on metal molds. The fs laser setup is shown in Figure 1. First, the laser beam is reflected by a galvanometer (Scanlab, SCANcube III 10) and focused by a field lens with a focal length of 160 mm and a focusing point of approximately 15 μm (1/*e*^2^). By moving the 3D translation stages, the fs laser beam can be focused on the surfaces of the 6061 aluminum alloys, and then the beam can ablate the samples at the focusing point. The focused point moves at a speed of 5 mm/s with a parallel line pattern on the aluminum alloy surfaces, and the interval space between the lines is a set value of 40 μm. After fs laser ablation, the gully structures with micro-nanostructures were made on the surfaces of the samples.

### 2.4 Imprinting

The thermal imprinting method is used to mimic the micro-nanostructures from the aluminum alloys to the PP sheets. Before imprinting, the molds are put into deionized water for 10 min of ultrasonic cleaning to remove the weakly deposited particles on the mold surfaces. Then, the molds are immersed in the saturated ethanolic stearic acid solution for 3 min to give the stearic acid full access into the structures, making it easy to release the PP sheets from the molds. After that, the molds are put on the heated lower platen of the compression press at a temperature of 120 °C. When the mold temperature reaches 120 °C, the PP sheets are put on the mold and then compressed by the heated upper platen. The pressure is from 0 to 20 MPa and is kept for 3 min. After that, the PP pieces are carefully released from the mold. The size of the aluminum alloys is only 5 × 5 cm^2^, so same step from imprinting to releasing of the different areas are required to be repeated several times for the whole surface of the trapezoid PP to have micro-nanostructures.

### 2.5 Rain Collection Test

**Figure 2:**
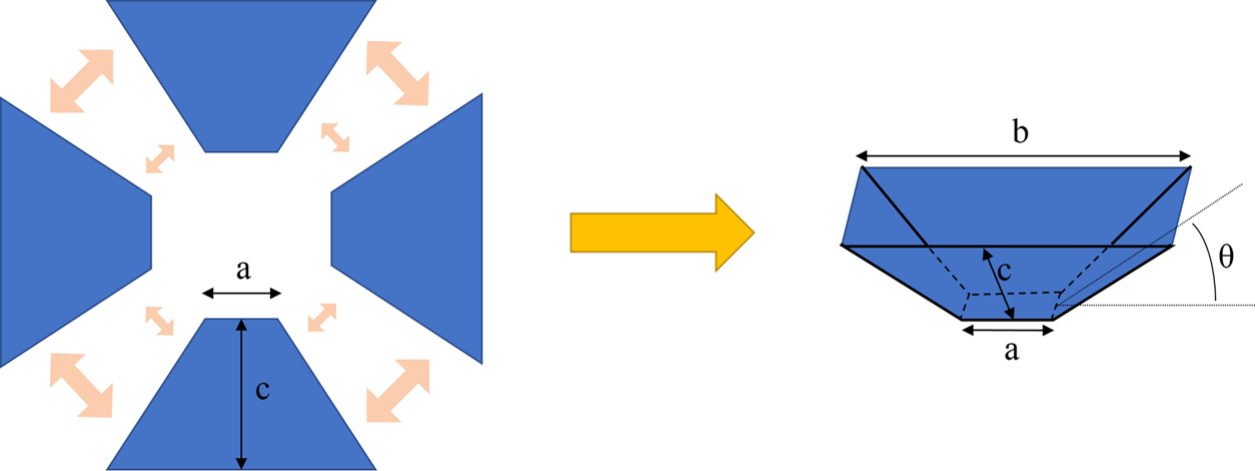
Rain collection funnel combined with trapezoid PP sheets.

**Figure 3:**
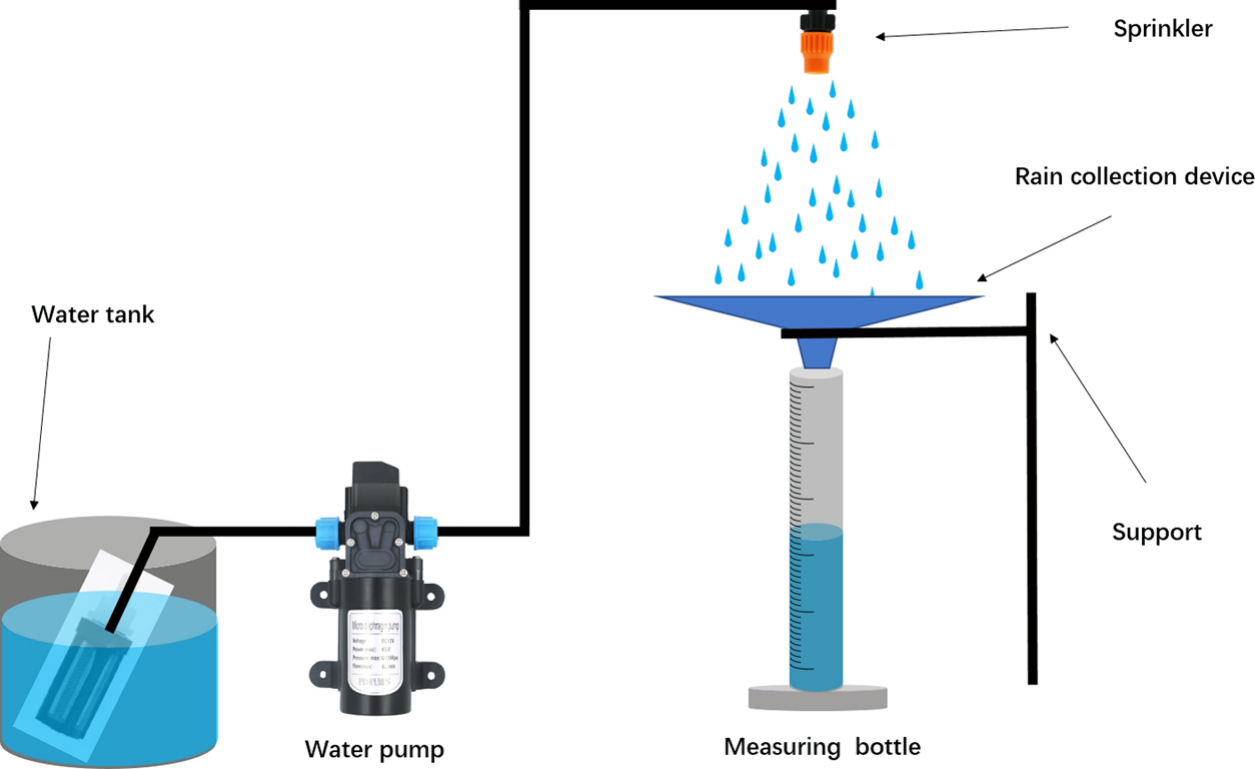
Schematic diagram for the investigation of rain collection efficiency. A sprinkler supported by a constant pressure water pump can generate the simulated rainfall.

Table 1 shows the parameters of the trapezoid PP sheets. The dimensions *a*, *b*, and *c* are the topline, baseline, and height of the trapezoid PP sheets, respectively. After imprinting, four trapezoid PP sheets with structures are combined to form the rain collection funnel, as shown in Figure 2. A modification of the angle θ can change the slope of the funnel’s sidewalls. The angle θ with values of 10, 30, 50, and 70° were created using different dimensions *c* with values of 34.5, 39.3, 52.9, and 99.4 mm, respectively. Additionally, the trapezoid dimensions *a* and *b* are fixed at the same values. The effective rainwater collection area of all of the rain collection funnels is *b* × *b*. Dimension c affects how many materials are needed to make the rain collection funnel, which affects the manufacturing cost. The more material is used, the greater the value of dimension *c* is, resulting in a greater value of the angle θ. The greater the angle θ, the higher the device is. Dimensions *a* and *b* are 12 and 80 mm, respectively. Furthermore, rain collection funnels without structures on the PP surfaces are made for comparisons. The spray system is made for testing the rain collection, as shown in Figure 2. A sprinkler supported by a constant pressure pump can generate the simulated rainfall shown in Figure 3. The raindrops randomly come from the sprinkler with a rough speed of 3 m/s and approximate diameters from 0.1 to 2 mm. The measuring bottle is put under the bottom of the rain collection funnel to the efficiency of the rain collection.

## 3 Results and Discussion

**Figure 4:**
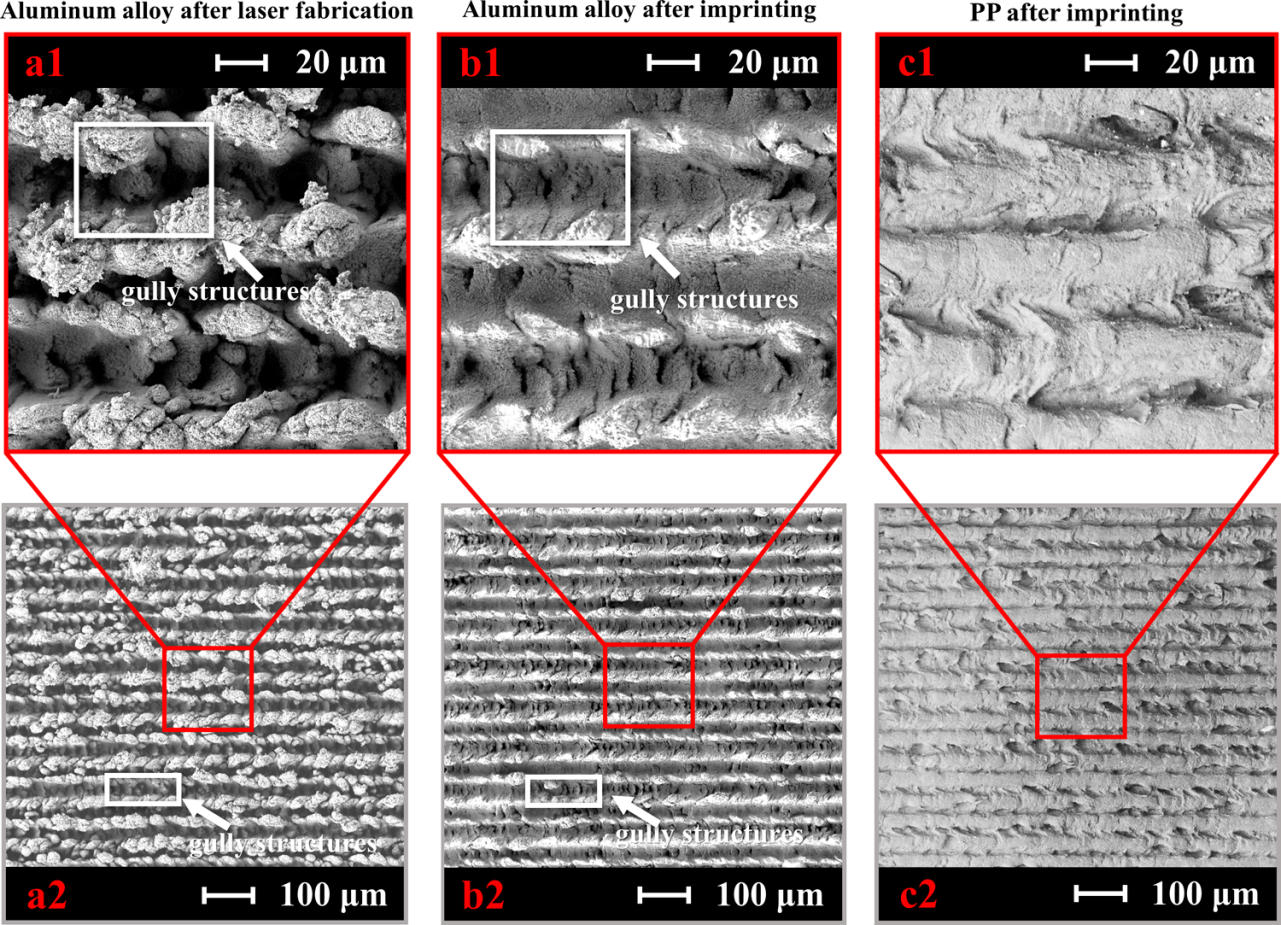
SEM images of (a1, a2) the aluminum alloy 6061 after fabrication and (b1, b2) after imprinting and (c1, c2) PP surface structure after imprinting.

**Figure 5:**
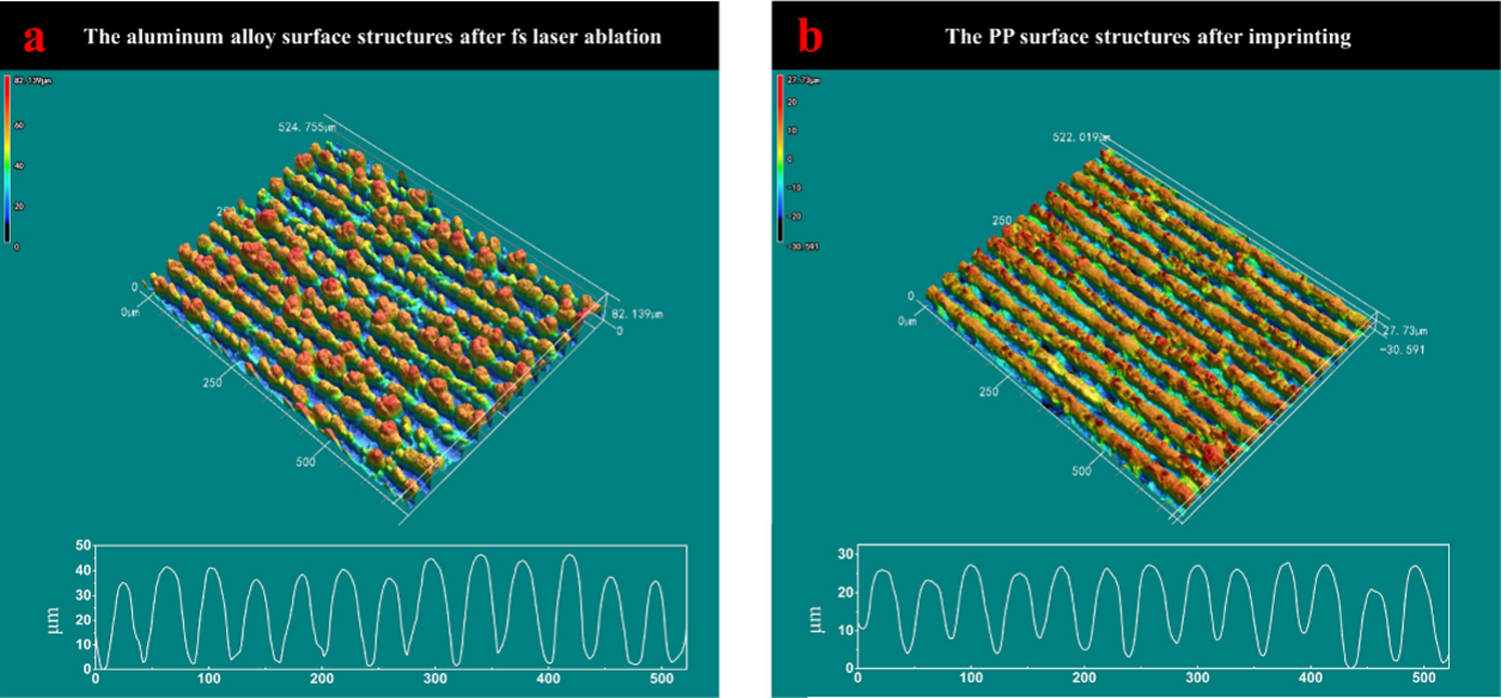
3D confocal images of (a) aluminum alloy 6061 after laser fabrication and (b) PP surface structures after imprinting.

The high power and frequency of the fs laser produces plenty of particles, some of which are deposited on the mold surface again and become irregular pellets from several hundred nanometers to dozen micrometers on the aluminum alloy surface, as shown in Figure 4. After the imprinting process, some not sturdy pellets get away with the aluminum surface and attach to the PP surface. The SEM picture of the PP Figure 4 shows that the nanoscale and microscale structures are copied from the alloy surface to the PP surface. The 3D confocal results in Figure 5 show that the aluminum alloy mold structures are about 40 μm in height, and the PP pieces after imprinting are also around 40 μm. Therefore, we can conclude that the structures are successfully copied from the mold to the PP surface with the SEM results. Interestingly, the aluminum composition is energy-dispersive spectrometry (EDS) detected on the PP surfaces, which means that some aluminum pellets moved to the PP surfaces, as shown in the Supporting Information Table S1. The aluminum alloy surface also changes due to loss of a few frail pellets; however, the aluminum alloy surface still has multiscale gully structures. The first few times imprinting from the same aluminum alloy mold to the PP surfaces have some aluminum pellets. After about ten imprinting times, the frail pellets on the aluminum alloy are nearly removed.

**Figure 6:**
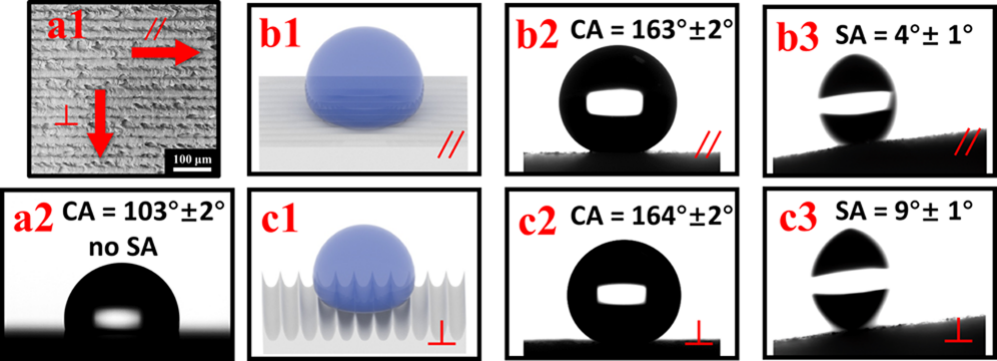
(a1) CAs are measured along with the parallel and perpendicular directions; (a2) is the CA measurement result with the plane PP (b1) and (c1) are the schematic pictures of the parallel and perpendicular of the CA and SA measurement of the imprinted PP surface; (b2) and (c2) are the CAs measurement results with parallel and perpendicular directions; (b3) and (c3) are the SAs measurement results with parallel and perpendicular directions.

After imprinting the structures on PP, we measure the contact angle (CA) and sliding angle (SA) in two perpendicular directions of the samples, one is parallel to the gully of the surface and the other perpendicular to the gully of the surface, as shown in Figure 6. The CAs are approximately more than 160° in both parallel and vertical directions, and the SAs are all smaller than 10°. The measured results mean that the samples have excellent superhydrophobic quality. To determine the wettability of the surface, the Cassie–Baxter (θ_CB_) equation is used
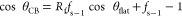
1The cos θ_CB_ is the contact angle of 163°, *R*_f_ is the roughness factor from the laser confocal microscope of 1.613, cosθ_flat_ is the contact angle of the flat surface of 105°, and *f*_s–1_ is the fraction of the solid surface area wetted by the liquid. By eq 1, the *f*_s–1_ can be calculated to be 0.075.^39,40^ The calculated result shows that 7.5% of the PP surface contacted the water, following the Cassie–Baxter model.

The CAs in both directions are nearly the same; by the way, the SAs of the parallel and vertical are much more different. While the SAs in the vertical direction are about 5–10°, those in the parallel direction are much smaller than 5°. This difference in the SAs can be attributed to the anisotropic nature of the surface structure. For the interline spacing of 40 μm, the differences of the CAs for different directions are not too much different, which has the same agreement with the research result of Florian et al.^41^ The reason is that the water droplets on the superhydrophobic surfaces are more likely to fall along the line’s path in the parallel direction than cross through the gully structures in the perpendicular direction of the surfaces. Therefore, this causes the SAs in the parallel direction to be smaller than those in the vertical direction. Based on this result, we make the gully structures parallel to the fall-down direction of the rain collection funnel.

**Figure 7:**
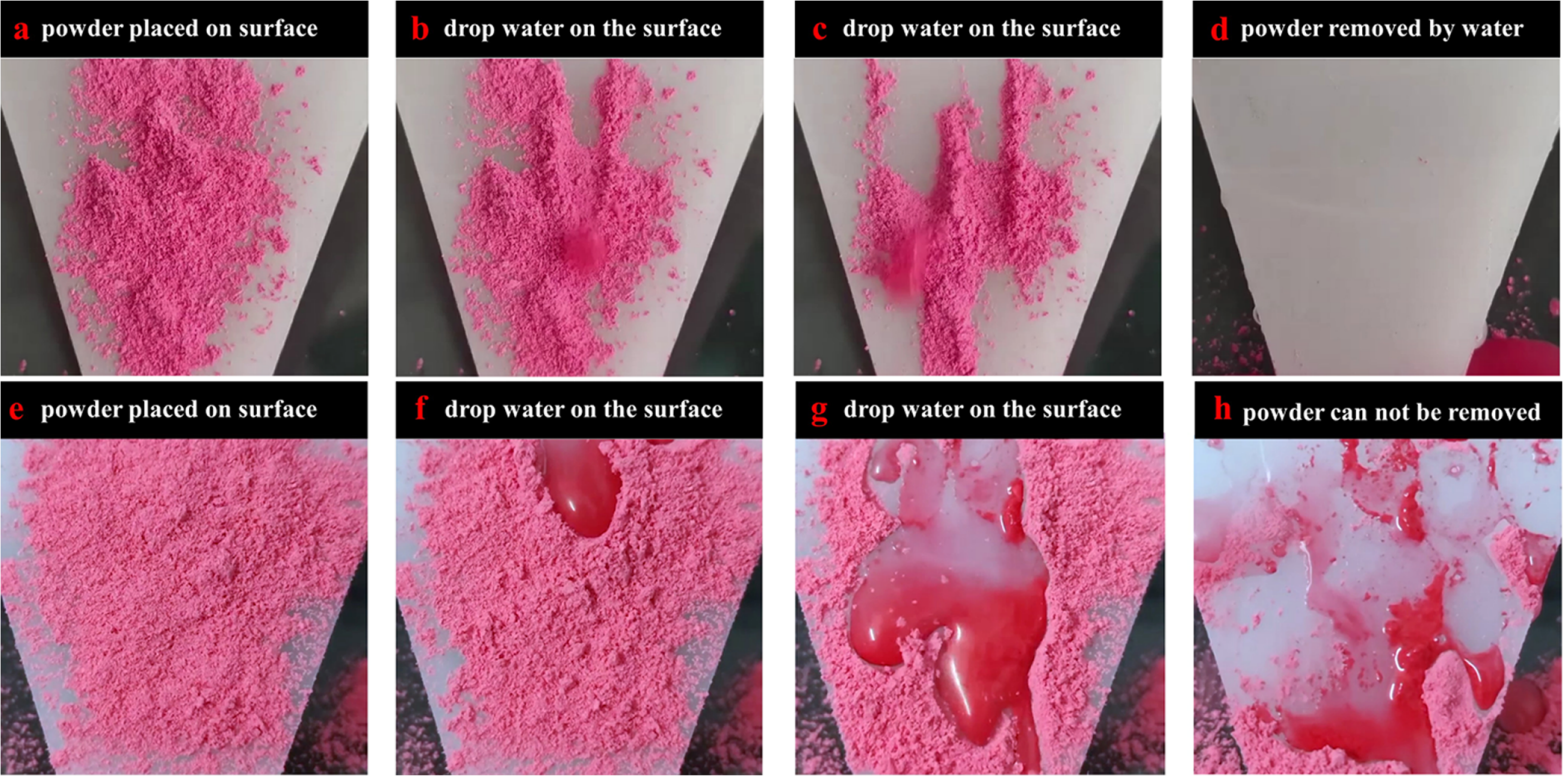
Self-cleaning test with powder on the imprinted (a–d) and untreated (e–h) PP surface: (a) powder particles are put on the imprinted surface; (b, c) water is dropped on the imprinted surface; (d) powder particles are removed out of the surface by water droplets; (e) powder particles are put on the untreated surface; (f, g) water is dropped on the untreated surface; and (h) powder particles cannot be removed out of the surface by the water droplets.

In this research, the superhydrophobicity formation can be explained by the replicated gully structures on the PP polymers and their low surface energy, which has the same agreement with the Wenzel theory.^39^ Moreover, the anisotropic property in sliding movement depends on the direction of microgrooves. Therefore, the superhydrophobic PP surfaces have self-cleaning properties. One big piece with a dimension *c* of 99.4 mm was produced after several imprinting cycles. The whole surface of the PP had micro-nanostructures. Moreover, lots of powder made by chalk is put on the PP surface to simulate the dust on the imprinted surface. Most of the particle sizes of powders in Figure 7 are between 5 and 30 μm. The powder covers the surface before dropping water on the PP surface, as shown in Figure 7a. After several water droplets are dropped on the surface, the water droplets remove the powder. In Figure 7b,c, the color of the water droplet is changed to the same color as the powder because the water droplets attach and remove the powder together after providing more water droplets on the surface. Finally, there is nearly no powder on the surface, as shown in Figure 7d. The obtained result means the imprinted PP surfaces have excellent self-cleaning properties. Figure 7 comes from the videos on the Supporting files Videos S1 and S2. The whole self-cleaning testing processing is shown in Video S1. As for comparison, an untreated PP sheet was used for the same self-cleaning test in Figure 7e–h. The powder on the surface cannot be removed easily by water droplets, and even though lots of water droplets attaching powder are on the untreated surface.

**Figure 8:**
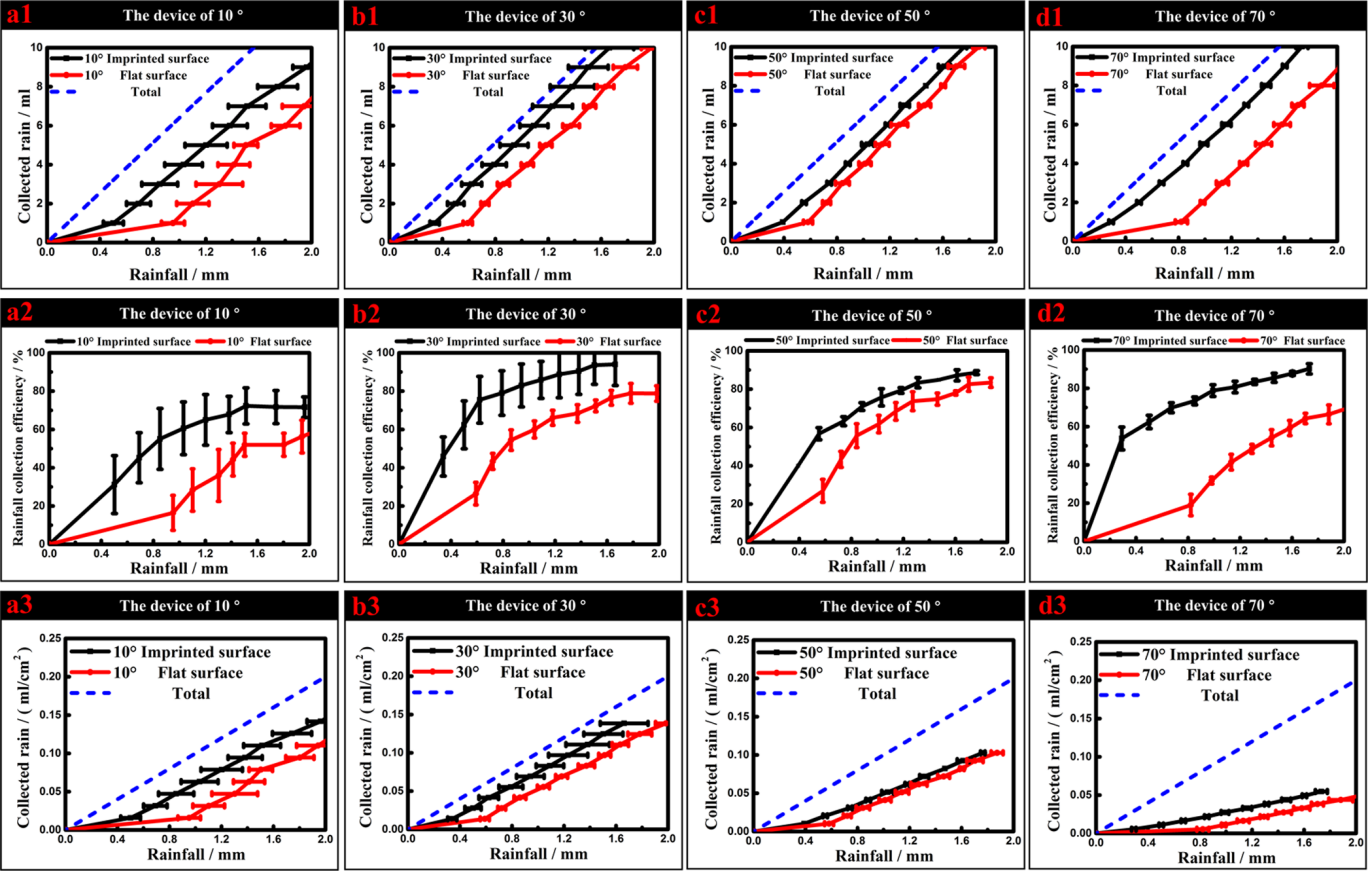
(a1–d1) Volume of the rain directly collected by the rain collection devices, (a2–d2) rain collection efficiency, and (a3–d3) material utilization of the flat and imprinted surface with different angles θ.

**Figure 9:**
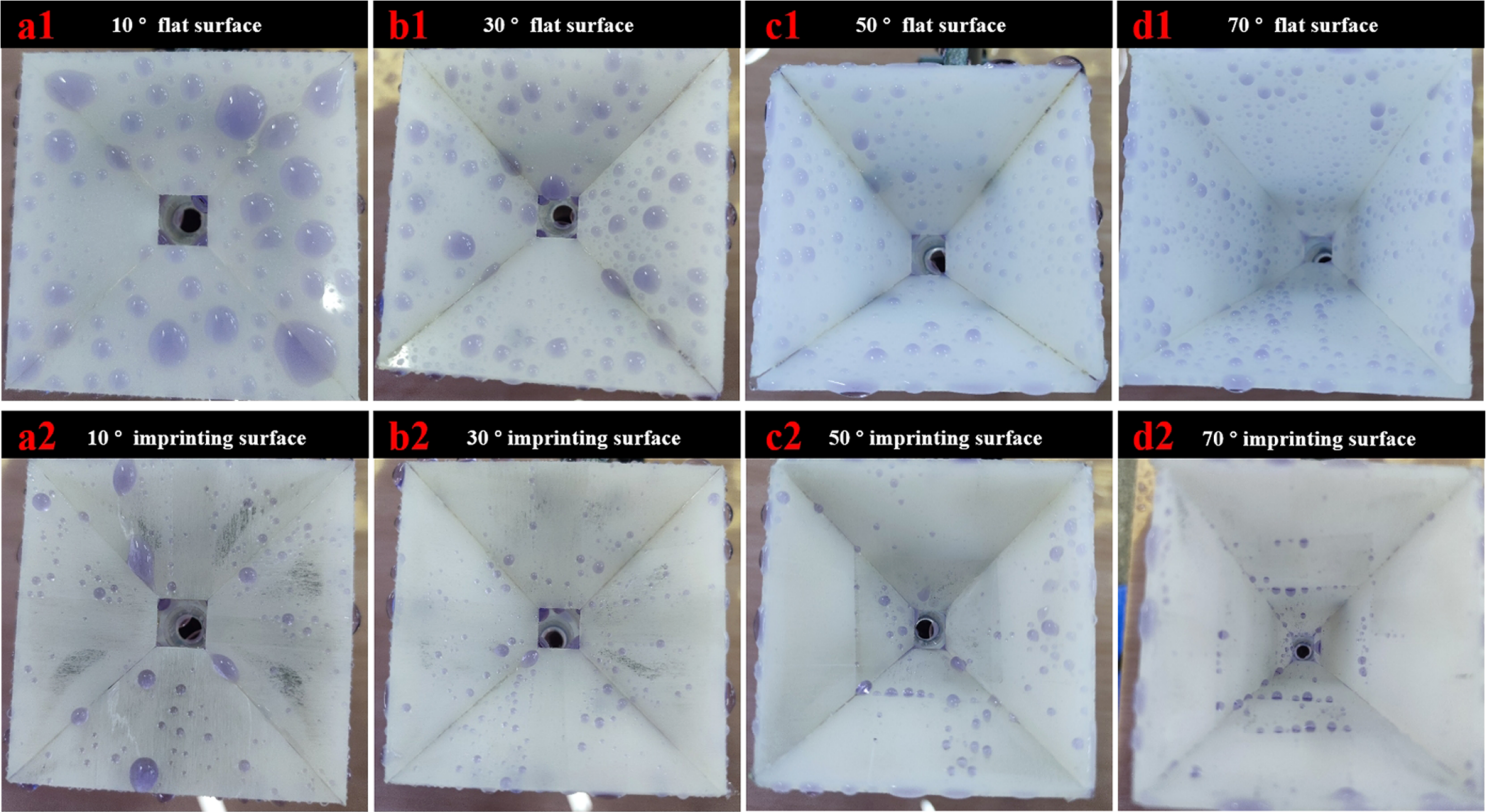
(a1), (a2), (a3), and (d1) devices combined by flat PP surfaces at different angles θ of 10, 30, 50, and 70°, respectively. (a2), (b2), (c2), and (d2) devices combined by imprinted PP with micro-nanostructures at different angles θ of 10, 30, 50, and 70°, respectively.

The imprinted surfaces are combined to form the small rain collection funnel device to test the rain collection effect. Each device made up of flat or imprinted surfaces with a different angle θ is used to collect the rain made by the rain collection device, and the testing results are then shown in Figure 8a1–d1. The data are three-time testing results, and the maximum and minimum data are used to make the error bar. Compared to the blue dot line (total rain made by the testing device), the device with the angle θ of 30° collected the most rain, as shown in Figure 8b1. It can be seen that with 0–2 mm rainfall, all of the imprinted gully-structured rain collection funnels with different angles θ can collect more water than the device made of flat surfaces, as shown in Figure 8a1–d1.
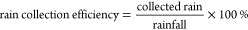
2The rain collection efficiency is calculated by the ratio between the collected water and the total water produced by the rain collections device, as shown in eq 2. It can be seen how the rain collection efficiency changed with the rain increasing from 0 to 2 mm based on Figure 8a2–d2. For the imprinted gully-structured rain collection device with the angle θ of 10°, the rain collection efficiency can reach about 60% when the rain is greater than 1 mm, whereas the efficiency of the flat surface without any structures is about 28%. Moreover, in the case of the angle θ of 30°, the flat surface can only collect about 26% of the rain when the rain is 0.6 mm. Under same conditions, for the angle θ of 30°, the rain collection efficiency of the imprinted gully-structured rain collection device can reach about 75% when the rain is greater than 0.6 mm. It means that about 75% of the raindrops on the rain collection device can be collected, more than 2 times higher than the surface without any structures. Especially when the rain is 1.6 mm, the rain collection efficiency for the angle θ of 30° can reach the maximum value of 90%. For the angle θ of 50°, the imprinted surfaces also have higher rain collection efficiency than the flat surfaces. At the same time, for the angle θ of 70°, the efficiency of the imprinted surfaces reaches about 70% at 0.8 mm of rain size, while the efficiency of the flat surfaces is only around 20%. This is because, for the angle θ of 70°, the flat surfaces hold more water droplets, as shown in Figure 9d1, and have more surface area. As a result, the efficiency of flat surfaces is much lower than that of the device with imprinted surfaces at the same angle. Moreover, the flat-surface device with an angle of 70° has lower efficiency than those with the angles θ of 30 and 50°. The flat-surface device with the angle θ of 10° has the lowest efficiency. The water droplets stick on the surface and do not easily fall from the surface, as shown in Figure 9a1. From Figure 8a2–d2, we can conclude that at the beginning, the raindrops stick on the surface and the flat surfaces hold much more water than the superhydrophobic gully structure surfaces.

For considering the material utilization, the “rain collected by unit surface area” is used to clarify the rain collection efficiency of the device with different angles θ in Figure 8a3–d3. “Rain collected by unit surface area” means how much water can be collected with the same amount of the materials, using the collected water divided by the surface area, as shown in eq 3. By comparing the “Rain collected by unit surface area”, it is clear that the “Rain collected by unit surface area” of devices with imprinted surfaces are higher than those of the flat surface for different angles θ. When the angle θ increases, the values of the “Rain collected by unit surface area” of both the flat and imprinted surfaces decreases, especially at the angle θ of 30 and 70°. Furthermore, the “rain collected by unit surface area” of the imprinted-surface device with the angle θ of 30° is a little higher than that with the angle θ of 10°.
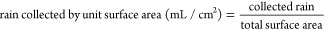
3In Figure 9, after the rain collection test, lots of water is stuck on the flat surfaces of the rain collection devices. To enhance the visibility of instinctively transparent water, the water is dyed to characterize how much water sticks on the devices. The gravity of water droplets increases when the angle θ increases. As a result, the bigger size of rainwater allows it to slide down from the surface. Moreover, the rainwater size on the flat surface decreases as the angle θ increases. In contrast, rainwater finds it more more challenging to attach to the surfaces of the rain collection devices made by superhydrophobic surfaces due to the low surface energies with micro-nanostructures. Therefore, only some small water droplets are seen on the superhydrophobic surfaces. In this study, the sizes of the PP sheets are larger than the molds’ sizes, so superhydrophobic surfaces show some boundary lines, causing attachment of some small rainwater droplets. Obviously, at the tilt angle of 70°, the devices with flat surfaces have the maximum water sticking to them. In other cases of the tilt angles, the devices with flat or structured surfaces have only some water droplets sticking to them, as shown in Figure 9. The water sticks on the whole surfaces of the flat surfaces and on some of the boundary areas of the imprinted surfaces. Moreover, the water on the surfaces with gully structures slips down effortlessly compared to the cases of the flat surfaces. Furthermore, the efficiencies of the imprinted gully-structured rain collection funnels are much higher than those of the flat-surface rain collection funnels. Interestingly, when the rainfall is from 0 to 1 mm, the efficiencies of the gully-structured surfaces at the angles of 10 and 70° are about 2–3 times higher than those of the flat surfaces. It means that for the slight rain, the surfaces with superhydrophobic gully structures can collect much more water than the flat surfaces. The efficiency increases when the rainfall increases because the water sticking on the surface reaches the saturation state, and more raindrops on the surface can be collected. Therefore, the imprinted structures can collect much more water than the flat surface even in case of light rain.

The results of the above experiments show that the imprinted gully-structured rain collection devices with the angles of the sidewalls at 10 and 30° can collect more water with less material requirement. Based on the rain collection results, the rain collection efficiencies of the devices formed by the superhydrophobic surfaces with the angles of the sidewalls at 10° extremely rapidly increase for the light rain compared to those formed by the flat surfaces. When the angle θ increases, the rain collection efficiency also grows, but more materials are needed. Moreover, when considering the size and cost of a large device, the angle θ of 10° is a good choice. However, if the rain collection efficiency is considered, the angle θ of 30° can be more suitable.

Based on the obtained results from the small-scale rain collection device, a big rain collection device of 50 cm × 50 cm with θ of 10° is made, which can be used for a sanitation system to collect the rain, exceptionally light rains in the areas of physical water scarcity, for cleaning purposes. The whole surface of the rain collection platform is covered by the 5 cm × 5 cm superhydrophobic imprinted PP sheets (Supporting Information Figure S1a1). Even though the rain droplets can attach to the surfaces at the boundaries of the PP sheets and the connecting areas, the flat surfaces attach more water than the superhydrophobic surfaces resulting in lower water collection efficiency (Supporting Information Figure S1a2,b2). In the future, an extensive mold can be made to produce large-scale superhydrophobic surfaces instead of connecting small PP sheets to optimize the current rain collection efficiency on the large-scale rain collection device for commercialization and practical applications in arid areas.

## 4 Conclusions

The gully structures were fabricated on the aluminum alloy mold by fs laser ablation and then imprinted to the PP sheets to manufacture self-cleaning superhydrophobic micro-nanogully structures. As a result, the superhydrophobic PP surfaces had excellent superhydrophobicity with the contact angles (CAs) greater than 160° and the anisotropic sliding angles (SAs) smaller than 5° in parallel directions and smaller than 10° in the vertical directions. The superhydrophobic formation is explained by a combination between micro-nanogully structures and their low surface energy. Moreover, different angles of the rain collection device were made to test the rain collection efficiency. When the rain is 1.6 mm, the collection efficiency of the device with the angle θ of 30° can reach the maximum value of 90%. Moreover, the rain collection device formed by the imprinted PP surfaces has higher rain collection efficiency per square centimeter compared with the one formed by the flat PP surfaces. The studied effects of the rain collection efficiency and total surface area showed that a rain collection device should have the sidewall at 10° angle for compact size and cost-saving and 30° angle for high rain collection efficiency, depending on the usage purposes. These data will help manufacture rain collection devices mainly applied to arid and drier countries and regions.

## Supporting Information Available

Additional table of the EDS results of the imprinting PP sheets and the figure for the big rain collection device testing results (PDF)
Whole self-cleaning testing processing of the imprinting PP sheets (Video S1) (MP4)
Whole self-cleaning testing processing of the flat PP sheets (Video S2) (MP4)
