# Supplementary material for: Fabrication of Superhydrophobic Gully-Structured Surfaces by Femtosecond Laser and Imprinting for High-Efficiency Self-Cleaning Rain Collection
Source: Langmuir. 2022 Feb 16;38(8):2720–8. doi: 10.1021/acs.langmuir.1c03488 (PMC9671392; doi:10.1021/acs.langmuir.1c03488)
Supplement: Supplementary file 1 — la1c03488_si_001.pdf [file la1c03488_si_001.pdf]

Fabrication of superhydrophobic gully-structured surfaces by femtosecond laser and imprinting for high-efficiency self-cleaning rain collection

Gan Yuan,[a,b] Yu Liu,[a,b] Fei Xie, [a,b] Chunlei Guo,[c] Chi-Vinh Ngo[a, *], Wei Li [a,b*]

[a] *GPL Photonics Lab, State Key Laboratory of Applied Optics, Changchun Institute of Optics, Fine Mechanics and Physics, Chinese Academy of Sciences, 130033, Changchun, China*

[b] *University of Chinese Academy of Sciences, 100049, Beijing, China*

[c] *The Institute of Optics, University of Rochester, Rochester, NY, 14627, USA*

**Corresponding authors: Chi-Vinh Ngo: chivinh@ciomp.ac.cn;*

Wei Li: weili1@ciomp.ac.cn.

supplemental document

Table S1 the EDS results of the PP surface after imprinted.

Element Number	Element Symbol	Element Name	Atomic Conc.	Weight Conc.	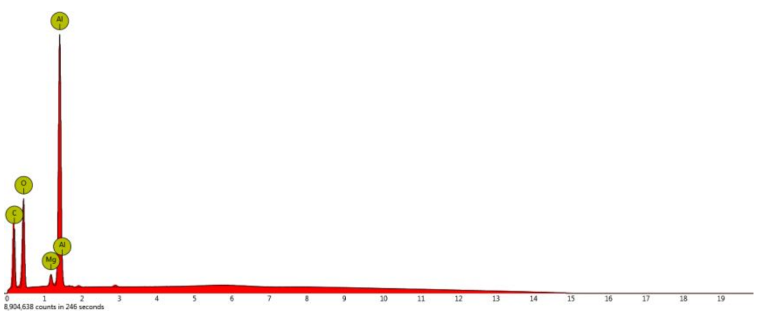
8	O	Oxygen	55.35	47.01	
13	Al	Aluminium	29.27	41.93	
6	C	Carbon	13.46	8.58	
12	Mg	Magnesium	1.92	2.48	

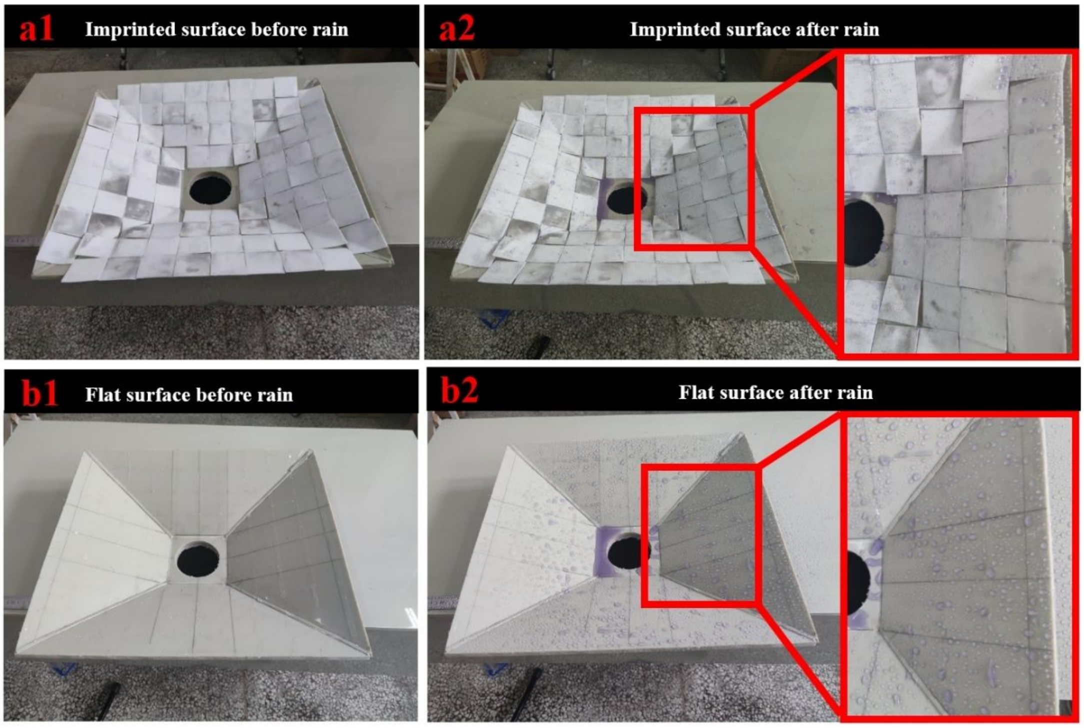

Fig. S1 (a1) and (b1) are the rain collection devices of 50cm*50cm with θ of 10°covered by imprinted superhydrophobic PP sheets and normal flat surface. (a2) and (b2) are the device covered by imprinted superhydrophobic PP sheets and normal flat surface after rain collecting test.
